# Trifecta Outcomes After Use of 3-Dimensional Digital Models for Planning of Robotic Prostatectomy: A Secondary Analysis of a Randomized Clinical Trial

**DOI:** 10.1001/jamanetworkopen.2024.34143

**Published:** 2024-09-16

**Authors:** Joseph D. Shirk, Robert E. Reiter, Eric M. Wallen, Raymond W. Pak, Thomas Ahlering, Ketan K. Badani, James R. Porter

**Affiliations:** 1 Department of Urology, David Geffen School of Medicine, University of California, Los Angeles; 2 Department of Urology, University of North Carolina School of Medicine, Chapel Hill; 3 Department of Urology, Mayo Clinic, Jacksonville, Florida; 4 Department of Urology, University of California, Irvine; 5 Department of Urology, Icahn School of Medicine at Mount Sinai, New York, New York; 6 Swedish Medical Center, Seattle, Washington

## Abstract

**Importance:**

Planning complex operations such as robotic-assisted laparoscopic radical prostatectomy (RALP) requires surgeons to review 2-dimensional magnetic resonance imaging (MRI) scans to understand 3-dimensional (3D) patient anatomy. Three-dimensional digital models for planning RALP may allow better understanding of patient anatomy and may lead to better patient outcomes, although data are currently limited.

**Objective:**

To determine surgical outcomes after RALP when surgeons reviewed 3D digital models during operative planning.

**Design, Setting, and Participants:**

This study was a planned secondary analysis of a multicenter, single-blind, randomized clinical trial conducted at 6 large teaching hospitals in the US. The study was conducted between January 1, 2019, and December 31, 2022, and included patients undergoing RALP. Patients were assessed and recruited at the time of surgical consultation. Final data analysis was conducted between August and December 2023.

**Intervention:**

Patients were randomized to either a control group undergoing usual preoperative planning with prostate biopsy results and multiparametric MRI only or to an intervention group in which imaging and biopsy results were supplemented with a 3D digital model. This model was viewed on the surgeon’s mobile phone in 3D format and picture-in-picture on the robotic console screen.

**Main Outcomes and Measures:**

The primary outcome measure for the overall study was oncologic outcomes after RALP, measured as prostate-specific antigen (PSA) detectability. Secondary outcomes were sexual function and urinary function, measured with Sexual Health Inventory for Men (SHIM) scores and rates of urinary incontinence, respectively, as well as use of salvage or adjuvant radiation therapy (RT) or androgen deprivation therapy (ADT). Trifecta outcomes were defined as undetectable PSA without RT or ADT, SHIM score categorically the same or greater than preoperatively, and complete continence. Univariate analysis was performed to compare outcomes between groups.

**Results:**

This trial included 92 patients undergoing RALP (51 in the control group and 41 in the intervention group). Their mean (SD) age was 62 (7.4) years; 10 patients (10.9%) were Black and 67 (72.8%) were White. At 18 months postsurgery, the intervention group had lower rates of biochemical recurrence (PSA level >0.1 ng/mL, 0 vs 7 [17.9%]; absolute difference, 17.9% [95% CI, 1.8% to 31.8%]; *P* = .01) and were significantly less likely to undergo adjuvant or salvage RT (1 [3.1%] vs 12 [31.6%]; absolute difference, 28.5% [95% CI, 10.1% to 46.7%]; *P* = .002) compared with the control group. Sexual function at 18 months postsurgery was significantly better in the intervention group (mean [SD] SHIM score, 16.8 [8.7] vs 9.8 [7.7]; absolute difference, 7.0 [95% CI, 2.6 to 11.4]; *P* = .002) and urinary function was unchanged (total continence, 22 [78.6%] vs 29 [80.6%]; absolute difference, 2.0% [95% CI, −17.9% to 21.9%]; *P* = .84) compared with the control group. Trifecta outcomes were achieved for 12 (48.0%) patients in the intervention group and 3 patients (10.0%) in the control group (absolute difference, 38.0% [95% CI, 14.4% to 61.6%]; *P* = .002).

**Conclusions and Relevance:**

In this secondary analysis of a randomized clinical trial, patients whose surgical planning of RALP involved 3D digital models had better oncologic and functional outcomes. Further work should assess the effect of 3D models in a broader set of patients, physicians, and hospital settings.

**Trial Registration:**

ClinicalTrials.gov Identifier: NCT03943368

## Key Points

**Question:**

Does the use of 3-dimensional (3D) digital models for planning of robotic-assisted laparoscopic radical prostatectomy (RALP) improve trifecta (ie, oncologic, sexual, and urinary) outcomes?

**Findings:**

In this secondary analysis of a randomized clinical trial with 92 patients undergoing RALP, the use of 3D digital models was associated with improved oncologic outcomes and sexual function without compromising urinary function.

**Meaning:**

In this study, 3D digital models allowed for better cancer control while improving functional outcomes in patients undergoing RALP.

## Teaser

This secondary analysis of a randomized clinical trial examines the effects of using 3-dimensional (3D) digital models during operative planning of robotic-assisted laparoscopic radical prostatectomy on oncological, sexual, and urinary outcomes.

## Introduction

Prostate cancer is the most common noncutaneous cancer in men, with more than 288 300 cases newly diagnosed each year; it is the second leading cause of cancer death in US men, trailing only lung cancer.^1,2^ Appropriate treatment of prostate cancer at a localized stage is critical to prevent future morbidity and potential death.^2^ Robotic-assisted laparoscopic radical prostatectomy (RALP) is a standard-of-care surgical operation that is accompanied by the risk of nononcologic, or functional, adverse effects that affect patient quality of life after treatment.^3,4^ Surgeons performing these operations are tasked with maximizing cancer control while preserving sexual function and urinary continence, known as trifecta outcomes, which may be achieved as low as 10% to 20% of the time in older men with higher-risk prostate cancer.^5,6,7^

Multiparametric magnetic resonance imaging (mpMRI) of the prostate has gained traction as a means to delineate anatomy and identify potential areas of prostate cancer.^8,9^ For prostate biopsy, the current standard of care involves mpMRI that is fused over a live ultrasound image, allowing targeting of areas concerning for prostate cancer.^10,11^ Trifecta outcomes depend on the surgeon’s precise understanding of the patient’s anatomy, particularly the relationship between the cancerous lesion (or lesions) and the capsule of the prostate, the bladder, and the nerves providing sexual and urinary function. Multiparametric MRI is substantially less valuable for surgical planning because of not only the difficulty reading these complex scans but also the relatively poorly defined visual anatomy in the scans.^12,13^ Three-dimensional (3D) digital imaging for surgical planning has recently been shown to improve understanding of patient anatomy, influence surgical plans, and improve patient outcomes.^14,15,16^ These effects may be due to an increased understanding of patient anatomy when viewed via a 3D model compared with 2-dimensional (2D) patient scans, which is especially critical when performing a complex operation using difficult-to-read imaging.

In this context, we identified patients undergoing RALP and performed a randomized clinical trial using 3D digital models generated from preoperative mpMRI scans. We sought to determine whether the use of patient-specific 3D models for RALP operative planning would affect trifecta outcomes. We previously reported the operative 3- to 6-month outcomes of these patients,^17^ in which we observed lower rates of postoperative prostate-specific antigen (PSA) detection and stable nononcologic outcomes when using the 3D model. Here, we report the 18- to 24-month trifecta outcomes of these patients.

## Methods

### Trial Design and Oversight

This is a secondary analysis of a multi-institution, single-blind randomized clinical trial, with enrollment and data collection occurring between January 1, 2019, and December 31, 2022. The study design was reported previously^17^ and the trial protocol and statistical analysis plan are presented in Supplement 1. Patients were assessed and recruited at the time of surgical consultation from 6 large US teaching hospitals in California (University of California, Los Angeles; and University of California, Irvine), Florida (Mayo Clinic), New York (Mount Sinai), North Carolina (University of North Carolina), and Washington State (Swedish Medical Center and Swedish Urology). The study protocol was approved by the Western Institutional Review Board and by the local institutional review board at 2 sites (Mount Sinai and University of North Carolina) and followed local regulations and the principles of the Declaration of Helsinki,^18^ the Council of International Organizations of Medical Sciences International Ethical Guidelines, and Good Clinical Practice guidelines. Written informed consent was obtained from patients at the time of enrollment. The study followed the Consolidated Standards of Reporting Trials (CONSORT) reporting guideline.

### Participants, Randomization, and Blinding

**Figure 1.**
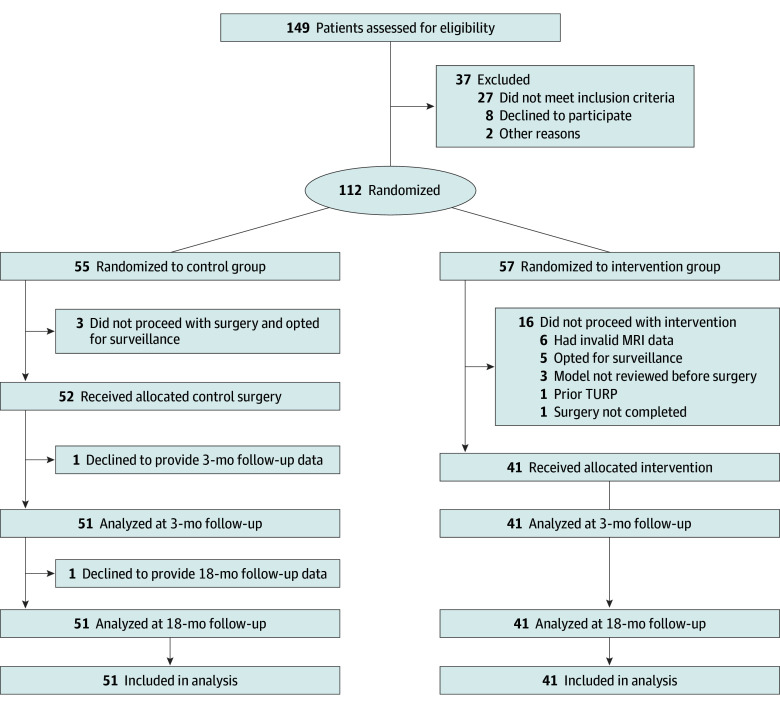
Flow Diagram of a Trial of 3-Dimensional Digital Models for Surgical Planning of Robotic-Assisted Laparoscopic Radical Prostatectomy MRI indicates magnetic resonance imaging; TURP, transurethral resection of the prostate.

Eligible participants were randomized to the control group or the intervention group (Figure 1). For the control group, the RALP operation was planned with surgeon review of the biopsy results and mpMRI only. For the intervention group, the surgeon additionally reviewed a 3D model of the patient’s anatomy that was created from the mpMRI and biopsy results. Exclusion criteria were as follows: prior prostate surgery, ablation, radiation therapy (RT), or androgen deprivation therapy (ADT); lack of 3-T or 1.5-T preoperative MRI with an endorectal coil; or inability to give informed consent. Randomization for the 15 surgeons in the study was surgeon specific, stratified by surgeon experience, and in permuted blocks at a 1:1 ratio (control:intervention) per surgeon. These blocks were designed to eliminate clustering by surgeon. Patients were assigned to a study group at enrollment using sequentially numbered, opaque, sealed envelopes containing the treatment assignment and were blinded to group assignment.

### Intervention

**Figure 2.**
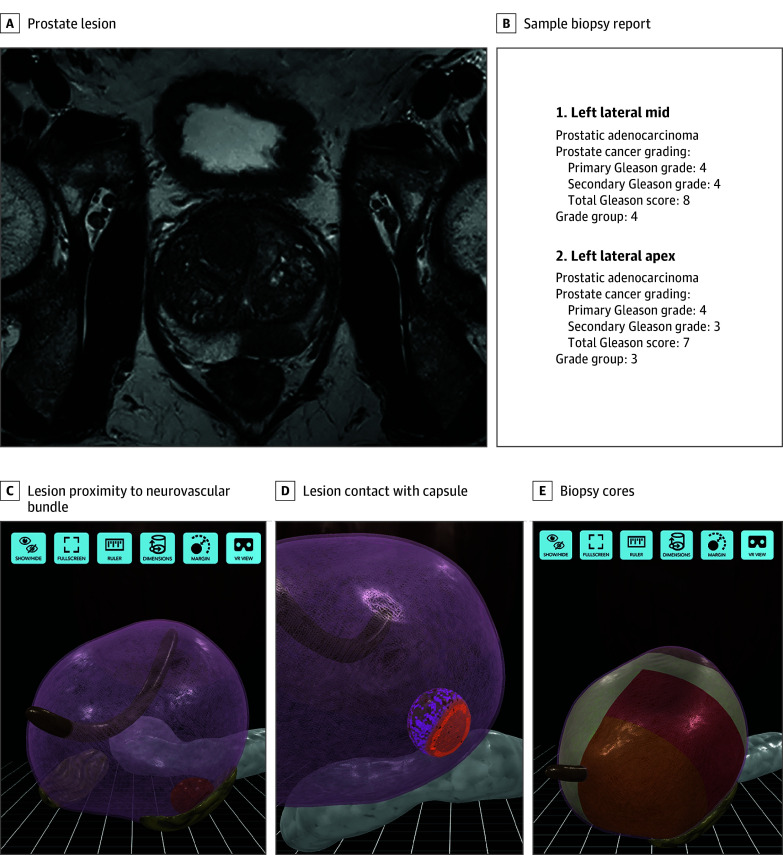
Magnetic Resonance Imaging and 3-Dimensional Digital Models of the Prostate A, Magnetic resonance image of a prostate with lesion. B, Sample patient biopsy report. C, Three-dimensional model of a prostate showing the proximity of the lesion (orange) to the neurovascular bundle (brown). D, Three-dimensional model with the neurovascular bundle hidden, showing wide contact of the lesion (orange) with the capsule (pink). E, Three-dimensional model of a prostate showing color-coded biopsy cores (Grade Group 3 is orange, and Grade Group 4 is red).

The mpMRI scans from the intervention group were deidentified and sent in Digital Imaging and Communications in Medicine format to Ceevra Inc, where they were used to create patient-specific 3D models (Ceevra Reveal, versions 2.4-2.8). The Prostate Imaging Reporting and Data System (PI-RADS) was used to identify and label prostate lesions. In addition, the biopsy report, which included grade group and location of biopsy cores, was used to create a patient-specific pathology map that was included in the 3D model. The 3D model included the prostate and capsule, lesion (or lesions), bladder, urethra, neurovascular bundles, seminal vesicles, and iliac vessels. The prostate and capsule were semitransparent, with delineation between them to facilitate identification of capsular bulging, extracapsular extension, or both. The bladder was semitransparent to allow inspection of the interface between the prostate and bladder (Figure 2).

The 3D models were delivered to a mobile application (Ceevra Reveal) installed on the surgeon’s smartphone, where they were viewed before and during surgery. Menus within the application contained patient-specific information, such as biopsy results, PI-RADS scores, and individual lesion size. The 3D model could be rotated and zoomed using standard touch-screen smartphone gestures, and the surgeon could show or hide each anatomic structure as well as view the 3D model picture-in-picture on the robotic console screen using a video cable connection between the smartphone and the surgical robot.

### Outcomes

Our primary outcome measure was oncologic outcomes after RALP. We defined each outcome measure at 3 time points: immediate postoperative, short term, and final assessment when oncologic and functional outcomes had reached a steady state. Oncologic outcomes included margin status, first postoperative PSA level (captured as early as 3 months but no later than 6 months postoperatively; hereinafter, 3-month follow-up), and follow-up PSA level (captured no earlier than 18 months and no later than 24 months postoperatively; hereinafter, 18-month follow-up), respectively. Initial PSA detectability was chosen as an indicator of poor prognosis, often leading to biochemical recurrence and future additional treatment, and was defined as greater than 0.1 ng/mL because most sites used standard PSA testing.^19^ In concordance with the latest American Urological Association and National Comprehensive Cancer Network guidelines and 2 recent randomized trials (GETUG-AFU 17 and RADICALS-RT), we defined biochemical persistence or recurrence at the 18-month time point as a PSA level greater than 0.1 ng/mL.^20,21,22,23^ To capture all adverse oncologic outcomes at the end of the study period, we also assessed whether patients had undergone adjuvant or early salvage RT or ADT (or both RT and ADT) by the 18-month follow-up.

The secondary end points were sexual and urinary outcomes. For sexual outcomes, nerve sparing status (a predictor of future erectile function) was captured immediately postoperatively.^24^ Sexual Health Inventory for Men (SHIM) scores were collected with PSA at 3 months and 18 months. For urinary outcomes, data on bladder neck sparing were collected immediately postoperatively, and the number of incontinence pads per day was captured at 3 months and 18 months. Eighteen months was chosen as the final assessment point for sexual and urinary function, because prior studies have shown little additional recovery in these domains after 18 months postoperatively.^25,26^ Trifecta outcomes were defined as an undetectable PSA level without RT or ADT, a SHIM score categorically the same or greater than preoperatively, and complete continence (0 incontinence pads per day).

Demographic data were collected for both groups from the medical records. Individuals were categorized, and race and ethnicity were self-reported (Asian, Black or African American, Hispanic or Latino, White, unknown, or declined to report) based on the National Institutes of Health Policy on Reporting Race and Ethnicity Data. Race and ethnicity were collected for descriptive purposes and not included in the statistical analysis.

Disease parameters collected included preoperative PSA level, grade group, lesion size and number, and PI-RADS score as well as postoperative pathological stage and grade group. Additional preoperative parameters included prostate size and preoperative SHIM scores. Clinical data collected included site, surgeon and surgeon experience level, and resident or fellow involvement in the surgery.

### Sample Size

Sample size was calculated using prior data, including previous trials using 3D models in robotic partial nephrectomy.^27^ We previously noted an effect size of 0.44 regarding the difference of outcome measures between the intervention (3D model–aided) and control groups. To account for multiple end points, we increased the sample size by 15%. Using the aforementioned method and the paired *t* test, the sample size of 90 (n = 45 per group) provided 80% power to detect a clinically significant 15% difference in rates of postoperative detectable PSA levels, with α = .05.

### Statistical Analysis

The analysis was modified intention to treat, because several patients did not receive treatment as randomized (exclusions were per protocol, and several patients in each group opted for other treatments or did not undergo appropriate preoperative imaging) (Figure 1). Missing data due to other mechanisms (eg, random nonresponse and data entry or collection) were addressed via pairwise deletion. As an initial analytic step, we compared baseline characteristics between cases performed with (intervention) or without (control) 3D models involved in preoperative planning. We used a 2-sample *t* test to compare means between groups and the chi-square test or Fisher exact test to assess the association between categorical variables. For the outcome measures, we used the appropriate aforementioned test to compare groups. To mitigate type I error due to multiple comparisons, a Bonferroni correction was used for the primary and secondary outcomes.

Two-sided statistical tests were performed using SAS, version 9.4 (SAS Institute); the significance threshold was set at *P* < .05. Final data analysis was conducted between August and December 2023.

## Results

### Study Participants

Of the 112 patients enrolled and randomized, 20 withdrew or were excluded (most commonly because they decided on treatments other than surgery or underwent an invalid MRI before surgery); therefore, 92 patients were included in the final analysis (Figure 1). The mean (SD) age of patients was 62 (7.4) years. In terms of race and ethnicity, 2 patients (2.2%) were Asian, 10 (10.9%) were Black, 3 (3.3%) were Hispanic, and 67 (72.8%) were White; 10 patients (10.9%) either declined to report their race or ethnicity or this information was unknown. Almost half of patients (40 [43.4%]) had high-risk disease. The mean (SD) preoperative PSA level was 8.4 (5.5) ng/mL, the mean (SD) prostate volume was 42.8 (19.9) mL, and the mean (SD) preoperative SHIM score was 18.6 (5.4) (Table 1). Surgeons performed the cases with standard robotic technique, with the only variation noted in seminal vesicle dissection approach (60% posterior).

**Table 1.** Baseline Characteristics Between Groups Who Underwent RALP With and Without 3D Digital Models^a^

Characteristic	Intervention group (n = 41)	Control group (n = 51)
Age, y		
Mean (SD)	61.7 (7.7)	62.7 (7.4)
Median (IQR)	63 (58-67)	64 (58-67)
Race and ethnicity		
Asian	2 (4.9)	0
Black	3 (7.3)	7 (13.7)
Hispanic or Latino	2 (4.9)	1 (2.0)
White	27 (65.9)	40 (78.4)
Unknown or declined to report	7 (17.1)	3 (5.9)
Preoperative PSA, mean (SD), ng/mL	8.2 (4.7)	8.6 (6.1)
Preoperative clinical stage		
T1c	10 (25.0)^b^	15 (29.4)
T2a/b/c	19 (47.5)	18 (35.3)
T3a	9 (22.5)	14 (27.5)
T3b	2 (5.0)	4 (7.8)
Preoperative grade group		
1-2	31 (75.6)	29 (59.2)^c^
3	2 (4.9)	4 (8.2)
≥4	8 (19.5)	16 (32.6)
Preoperative risk group		
Favorable intermediate	15 (36.6)	20 (39.2)
Unfavorable intermediate	9 (22.0)	8 (15.7)
High	17 (41.4)	23 (45.1)
Maximum PI-RADS score, 1-5		
Mean (SD)	4.1 (0.7)	4.2 (0.9)
Median (IQR)	4 (4-5)	4 (4-5)
Prostate volume, mL		
Mean (SD)	45.3 (22.4)	40.7 (18)
Median (IQR)	39 (28-53)	38 (28-53)
Preoperative SHIM score, mean (SD)	18.7 (7.4)	18.6 (5.4)
No. of RALP cases per year by surgeon experience		
1-10	3 (7.3)	3 (5.9)
11-30	5 (12.2)	7 (13.7)
≥31	33 (80.5)	41 (80.4)
Pathological stage		
T2	26 (63.4)	27 (52.9)
T3a	11 (26.8)	18 (35.3)
T3b	4 (9.8)	6 (11.8)
Postoperative grade group		
1-2	23 (62.2)^d^	32 (65.3)^c^
3	6 (16.2)	6 (12.2)
≥4	8 (21.2)	11 (22.4)

Abbreviations: 3D, 3-dimensional; PI-RADS, Prostate Imaging Reporting and Data Standards; PSA, prostate-specific antigen; RALP, robotic-assisted laparoscopic radical prostatectomy; SHIM, Sexual Health Inventory for Men.

^a^ Unless indicated otherwise, values are presented as No. (%) of patients.

^b^ Data missing for 1 patient.

^c^ Data missing for 2 patients.

^d^ Data missing for 4 patients.

### Primary End Point

For oncologic outcomes, positive margins were noted in 16 patients (32.7%) in the control group compared with 10 (25.0%) in the intervention group (absolute difference, 7.7% [95% CI, −11.3% to 26.7%]; *P* = .42; Table 2). The overall positive margin rate was 29%. The difference in positive margin rate between groups was greater in patients with higher-grade and higher pathological-stage disease but was not significant (eFigure in Supplement 2). The number of patients with detectable PSA levels was significantly lower in the intervention group relative to the control group at 3 months (1 [3.1%] vs 7 [19.4%]; absolute difference, 16.3% [95% CI, 1.3% to 31.5%]; *P* = .03) and at 18 months (0 vs 7 [17.9%]; absolute difference, 17.9% [95% CI, 1.8% to 31.8%]; *P* = .01).

**Table 2.** Comparative Outcomes Between the Intervention and Control Groups Who Underwent RALP With and Without 3D Digital Models

Outcome	Intervention group (n = 41)^a^	Control group (n = 51)^a^	Absolute difference, % (95% CI)	*P* value
**Oncologic **				
Margin status				
Negative	30 (75.0)	33 (67.3)	7.7 (−11.3 to 26.7)	.42
Positive	10 (25.0)	16 (32.7)		
PSA, ng/mL				
3 mo				
Undetectable	32 (96.9)	29 (80.6)	16.3 (1.3 to 31.5)	.03
Detectable	1 (3.1)	7 (19.4)		
18 mo				
Undetectable	32 (100)	32 (82.1)	17.9 (1.8 to 31.8)	.01
Detectable	0	7 (17.9)		
RT with or without ADT by 18 mo				
Yes	1 (3.1)	12 (31.6)	28.5 (10.1 to 46.7)	.002
No	31 (96.9)	26 (68.4)		
**Sexual **				
Nerve sparing				
None	5 (12.2)	3 (5.9)	6.3 (−5.3 to 18.0)	.43
Unilateral	4 (9.8)	8 (15.7)		
Bilateral	32 (78.0)	40 (78.4)		
SHIM score				
3 mo				
Mean (SD)	11.1 (9.4)	11.7 (7.1)	0.6 (−4.5 to 3.3)	.76
<17	21 (67.7)	25 (67.6)	0.1 (−22.5 to 22.2)	.99
≥17	10 (32.3)	12 (32.4)		
18 mo				
Mean (SD)	16.8 (8.7)	9.8 (7.7)	7.0 (2.6 to 11.4)	.002
<17	9 (36.0)	21 (70.0)	34.0 (7.5 to 60.4)	.01
≥17	16 (64.0)	9 (30.0)		
**Urinary**				
Bladder neck sparing				
Yes	33 (86.8)	40 (93.0)	6.2 (−6.8 to 19.2)	.35
No	5 (13.2)	3 (7.0)		
Pad usage				
3 mo				
Mean (SD)	0.97 (1.4)	1.4 (2.8)	0.43 (−1.4 to 0.5)	.38
0	22 (56.4)	23 (50.0)	6.4 (−14.9 to 27.7)	.56
≥1	17 (43.6)	23 (50.0)		
18 mo				
Mean (SD)	0.36 (0.8)	0.31 (0.7)	0.5 (−0.3 to 0.4)	.79
0	22 (78.6)	29 (80.6)	2.0 (−17.9 to 21.9)	.84
≥1	6 (21.4)	7 (19.4)		

Abbreviations: 3D, 3-dimensional; ADT, androgen deprivation therapy; PSA, prostate-specific antigen; RALP, robotic-assisted laparoscopic radical prostatectomy; RT, radiation therapy; SHIM, Sexual Health Inventory for Men.

^a^ Unless indicated otherwise, values are presented as No. (%) of patients.

### Secondary End Points

**Figure 3.**
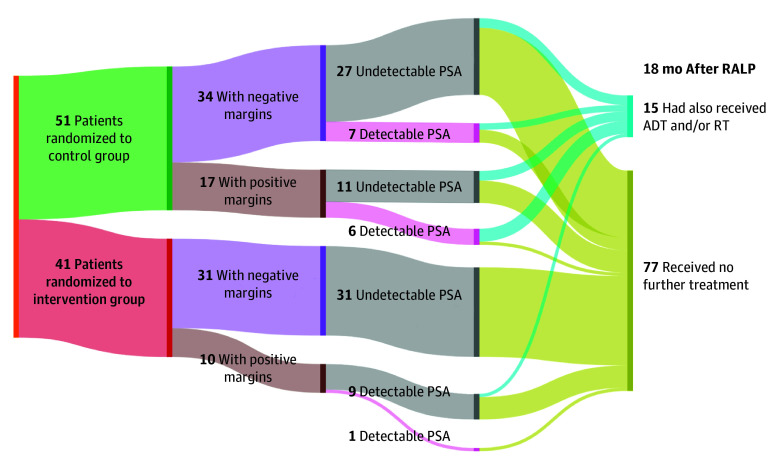
Study Flow by Group, Margin Status, Prostate-Specific Antigen (PSA) Detection (>0.1 ng/mL), and Postsurgical Treatment Patients (N = 92) were randomized to either a control group undergoing usual preoperative planning for robotic-assisted laparoscopic radical prostatectomy (RALP) with prostate biopsy results and multiparametric prostate MRI only or to an intervention group undergoing RALP in which imaging and biopsy results were supplemented with a 3D digital model. ADT indicates androgen deprivation therapy; RT, radiation therapy.

Patients in the intervention group were significantly less likely to undergo adjuvant or salvage RT by 18 months than those in the control group (1 [3.1%] vs 12 [31.6%]; absolute difference, 28.5% [95% CI, 10.1% to 46.7%]; *P* = .002). The flow of patients by group, margin status, detectable PSA level greater than 0.1 ng/mL, and postsurgical treatment is shown in Figure 3.

For sexual function, mean (SD) preoperative SHIM scores were similar between the intervention and control groups (18.7 [7.4] vs 18.6 [5.4]; absolute difference, 0.1 [95% CI, −2.8 to 2.6]; *P* = .92). A similar number of patients in each group underwent nerve sparing (36 [87.8%] vs 48 [94.1%]; absolute difference, 6.3% [95% CI, −5.3% to 18.0%]; *P* = .43) and among patients grouped by postoperative grade group and pathological stage (eFigure in Supplement 2). At 3 months, mean (SD) SHIM scores were also similar between the intervention and control groups (11.1 [9.4] vs 11.7 [7.1]; absolute difference, 0.6 [95% CI, −4.5 to 3.3]; *P* = .76). At 18 months, the intervention group had higher mean (SD) SHIM scores compared with the control group (16.8 [8.7] vs 9.8 [7.7]; absolute difference, 7.0 [95% CI, 2.6 to 11.4]; *P* = .002). The intervention group also had a higher percentage of SHIM scores greater than 17, which signifies no worse than mild erectile dysfunction (16 [64.0%] vs 9 [30.0%]; absolute difference, 34.0% [95% CI, 7.5% to 60.4%]; *P* = .01).

For urinary function in the intervention and control groups, there was no difference in bladder neck sparing (33 [86.8%] vs 40 [93.0%]; absolute difference, 6.2% [95% CI, −6.8% to 19.2%]; *P* = .35), 3-month mean (SD) number of pads per day (0.97 [1.4] vs 1.4 [2.8]; absolute difference, 0.43 [95% CI, −1.4 to 0.5]; *P* = .38), or 18-month mean (SD) number of pads per day (0.36 [0.8] vs 0.31 [0.7]; absolute difference, 0.5 [95% CI, −0.3 to 0.4]; *P* = .79). The rate of total continence (0 incontinence pads per day) was similar for the intervention and control groups at 18 months (22 [78.6%] vs 29 [80.6%]; absolute difference, 2.0% [95% CI, −17.9% to 21.9%]; *P* = .84).

### Trifecta Outcomes

A total of 12 patients (48.0%) in the intervention group achieved trifecta outcomes vs 3 patients (10.0%) in the control group. The absolute difference was 38.0% (95% CI, 14.4% to 61.6%) (*P* = .002).

## Discussion

Patients undergoing treatment for localized prostate cancer have multiple treatment options with similar oncologic efficacy. The differences among these options are the nononcologic adverse effects experienced after treatment, which have been well defined by long-term outcome data.^28,29^ When performing RALP for prostate cancer, surgeons must balance oncologic control with both sexual and urinary function, which are often dramatically affected after surgery. In this context, our study had several important findings.

When 3D models were used in this study, oncologic outcomes improved, with significantly lower detectable postoperative PSA levels and biochemical recurrence rates. These factors are highly predictive of the need for additional adjuvant or salvage therapy—most commonly, external beam RT with or without ADT.^23^ Not only is this treatment costly, it also confers additional adverse effects and morbidity.^23^ Additionally, these patients experience higher rates of metastasis and disease-specific mortality, with 30% or more developing metastatic disease.^30,31^ The 3D models used in this study may have allowed surgeons to better visualize the tumor and the relationship to the capsule in these complex cases, leading to a better oncologic plan. Although positive margin rates were only slightly lower overall with the use of 3D models, there was a larger effect for patients with higher-grade and higher-stage disease. This effect was likely the driver for the difference in PSA and lower progression to RT or ADT (or both) in patients for whom 3D models were used; however, the study was not powered to detect overall differences in margin status or for subgroup analysis. Additionally, the variability introduced by the lack of centralized pathology may have caused a discordance between true and reported positive margin rates.^32,33,34^

Sexual function was also better in patients undergoing RALP with 3D models. The neurovascular bundle critical for erectile function runs just lateral and posterior to the prostate, directly adjacent to the peripheral zone of the prostate, where most cancers are located. This is not a discreet structure but rather an assembly of nerve fibers and blood vessels that tightly traverse the surface of the prostate.^35^ We were only able to use binary quantification for nerve sparing (yes or no); in fact, each patient likely had a varying percentage of the neurovascular bundle preserved (referred to as partial nerve sparing). The 3D model, with a clearer depiction of the location of the lesion relative to the neurovascular bundle, may have led to a more individualized nerve sparing approach. The difference in SHIM scores likely reflects this, as do the higher rates of RT and ADT in the control group, which has significant effect on sexual function.

Although we noted improvements in oncologic and sexual outcomes, we did not note an effect of the 3D models on urinary outcomes. Had surgeons using the 3D models simply prioritized oncologic outcomes, taking wider margins to avoid leaving cancer behind, this could have negatively affected urinary outcomes through a reduction in bladder neck sparing and damage to the pelvic floor muscles.

The goal of RALP is to render the patient disease free while perfectly preserving their sexual and urinary function. Although this aim has traditionally been referred to as a trifecta, it is notoriously difficult to achieve, so much so that it has been suggested that the confluence of these outcomes should instead be referred to as a triple crown to signify 3 substantial wins for the patient in the context of their surgical outcomes.^36^ This is especially true in patients with high-risk disease, such as those in this study; with our very strict definitions for trifecta outcomes, the difference between the 2 groups is marked and highlights the convergence of outcome improvements seen when using the 3D models.^37^

The human ability to reconstruct 2D images into 3D images, as surgeons are tasked with doing when interpreting mpMRI scans, is limited.^38^ In addition, the cognitive load from nonpertinent elements of the patient anatomy on mpMRI scans restricts cognition during the surgical interpretation of imaging.^39,40,41^ The 3D models depict only the key structures relevant to the operation, while clearly representing tissue contours and junctions between tissues.^42^ With biopsy results included in the 3D model, the surgeon has all relevant details for the operation in one place for an integrated and intuitive review. We previously noted that surgeons in this study changed their operative plans around a third of the time when using the 3D models.^17^ The changes mostly concerned nerve sparing and surgical margins, which may have allowed surgeons to select the optimum operative pathway for each patient, optimizing cancer control when necessary, and allowing for preservation of sexual function when able.

### Limitations

This study has several limitations. The overall positive margin rate of 29% is at the upper end of the average reported in previous studies for this operation, which range from 6.5% to 32%.^43^ However, stage-specific rates vary; given that our cohort was composed of patients with higher-stage disease, our predicted positive margin rate was in the mid-20% range.^44^ Additionally, higher rates of RT, ADT, or both among patients in the control group likely affected their sexual function. However, as part of the broader picture, patients in the intervention group experienced better sexual outcomes as a result of better oncologic outcomes. Finally, our patient and surgeon populations were relatively homogenous.

## Conclusions

This secondary analysis of a randomized clinical trial explored the effects of patient-specific 3D models for RALP operative planning on trifecta outcomes. The results of this study suggest that novel forms of surgical planning (eg, those involving 3D models that represent the size, orientation, and proximity of these structures) may help improve surgeon understanding of patient anatomy and, as a result, lead to better outcomes for patients undergoing RALP. Further work should assess the effect of 3D models in a broader set of patients, physicians, and hospital settings.

## Notes

Supplement 1.
Trial Protocol and Statistical Analysis Plan

Supplement 2. **eFigure.** Positive Margin Rate Between Study Group by Grade Group and Pathological Stage

Supplement 3.
Data Sharing Statement
