# Supplementary material for: Trifecta Outcomes After Use of 3-Dimensional Digital Models for Planning of Robotic Prostatectomy: A Secondary Analysis of a Randomized Clinical Trial
Source: JAMA Netw Open. 2024 Sep 16;7(9):e2434143. doi: 10.1001/jamanetworkopen.2024.34143 (PMC11406400; doi:10.1001/jamanetworkopen.2024.34143)
Supplement: Supplement 1. — Trial Protocol and Statistical Analysis Plan [file jamanetwopen-e2434143-s001.pdf]

Protocol for Research Study
(WIRB Protocol Number 20171006)

"Using Virtual Reality (VR) Models for Preoperative Planning for RALP"

I. STUDY OBJECTIVES. To assess improvement in patient outcomes when using 3D models

II. TARGET OPERATION. Robotic Assisted Laparoscopic Radical Prostatectomy (RALP)

III. SITES AND PRINICPAL INVESTIGATORS

a. Dr. Robert Reiter, UCLA
b. Dr. James Porter, Swedish Hospital
c. Dr. Ray Pak, The Mayo Clinic
d. Dr. Eric Wallen, UNC
e. Dr. Thomas Ahlering, UCI
f. Dr. Ketan Badani, Mt Sinai

IV. STUDY DESIGN.

a. **Trial Design**: A multi-institution, randomized, single-blind clinical trial

b. **Participants and Exclusion Criteria:** Eligible subjects identified from the medical record will be those who are 1) scheduled to undergo RALP surgery, 2) willing to consent to the surgery being performed by one of the surgeons participating in the study, and 3) willing to be assigned by randomization to one of the two groups in the study. Exclusion criteria are 1) prior prostate surgery, ablation, radiation, or androgen deprivation therapy, 2) lack of 3 Tesla or 1.5 Tesla with endorectal coil preoperative MRI imaging, and 3) inability to give informed consent.

c. **Randomization:** Each case will be assigned to one of the two preparation methods by a randomization procedure that is stratified by surgeon. Each surgeon-specific randomization schedule will be prepared using permuted blocks of size 2; i.e., each 2-patient block will be some permutation of {VR-aided, Control}. Thus, each surgeon's cases will be randomized in a 1:1 ratio. Use of permuted blocks thus avoids any confounding of preparation method with temporal trends (e.g., trend due to a learning curve.)

Sequentially numbered opaque sealed envelopes (SNOSEs) will be prepared for each individual surgeon. Each envelope will contain the treatment assignment for one case (intervention or control). There will be an equal number of envelopes containing intervention assignments and control assignments; the total number of envelopes provided to each surgeon will be determined based on the surgeon's historical/anticipated case volume. The treatment code will remain concealed inside the envelope until the moment the case is ready to be prepared.

d. **Blinding:** Patients, personnel gathering data from the video review, and statistician will be blinded. Control and intervention groups identifiers will be known only to the research personnel opening the sealed envelope for random group assignment and extracting patient data from the electronic medical record.

e. **Intervention:** For intervention cases, surgeons will be provided with a 3D model to view (a) prior to the operation in addition to the MRI and biopsy results and (b) during the operation in addition to the MRI. For control cases, surgeons will view only the MRI and biopsy results prior to the operation and during the operation itself.

f. **3D Model Creation and Delivery:** MRI scans will be deidentified and sent in Digital Imaging and Communications in Medicine (DICOM) format to Ceevra, Inc., where a patient-specific 3D model for each intervention patient will be created. The Prostate Imaging-Reporting and Data System (PIRADS) score will be used to identify and label prostate lesions. In addition, the biopsy report, will be used to create a patient-specific pathology map that will be included in the model to assist the surgeon in understanding the location of biopsy-proven cancer within the prostate. Models will be delivered to a mobile application on the surgeon's smartphone (Ceevra Reveal) and viewed as described above. In addition, the surgeon may view the model picture-in-picture on the robotic console screen using a video cable connection between the surgeon's smartphone and the surgical robot.

g. **Outcomes Measures and Covariates:** The primary outcome measure is oncologic outcomes after RALP. This outcome will be measured at three time points: immediately postoperative, short term, and a final assessment when oncologic and functional outcomes have reached a steady state. Oncologic outcomes will be measured as margin status (positive or negative), PSA (linear and detectable/undetectable) at the initial assessment (which in most cases is three months postoperatively but may occur any time between 3 and 6 months), and PSA (>0.1) at the final assessment (to be collected after 18 months but no later than 24 months). Additionally, any use of androgen deprivation therapy or radiation therapy will be captured at 24 months as an adverse oncologic outcome.

Secondary outcomes are the urinary and sexual function of the patient, measured at the same three time points as above. For urinary function, this is bladder neck sparing (yes or no), measured immediately postoperatively, and incontinence pads per day, measured at both 3-6 months and 18-24 months postoperatively. For sexual function, this is nerve sparing (none, left, right, or bilateral), measured immediately postoperatively, followed by SHIM score, measured at both 3-6 months and 18-24 months postoperatively.

Demographic data, disease-specific, surgery-specific, and surgeon-specific covariates will be collected from the medical record as well, see Appendix A.

h. **Sample Size:** Sample size was calculated using prior data, including previous trials using 3D models in robotic partial nephrectomy. Previously we noted an effect size of 0.44 regarding the difference of outcome measures between 3D

model-aided and control groups. To account for multiple endpoints, we raised the sample size by 15%. Using the above method and paired t-test, the sample size of 90 (45 per arm) provided 80% power to detect clinically significant differences in our primary outcome measures with $\alpha=0.05$.

i. **Statistical analysis:** Initial analysis will compare baseline characteristics between cases performed with (intervention) or without (control) models involved in preoperative planning. A two-sample t-test will be employed to compare means between groups and Chi-Square test or Fisher's exact test to assess the association between categorical variables. For outcome measures, the appropriate test will be used as described above to compare groups. To mitigate type I error due to multiple comparisons, and if the randomization yields balanced cohorts, a Bonferroni correction may be utilized for the primary and secondary outcomes. If randomization yields groups with statistically or clinically significant differences, adjusted differences between groups will be studies using a linear mixed model to regress the endpoint variables on covariates listed above**,** or the logistic mixed model, as appropriate.

V. SURGEON SURVEYS

a. **Pre- and Postoperative Surgeon Surveys** will be administered to assess various factors, such as surgeon change in operative plan, model quality, and utility of the models. The preoperative survey will be administered for intervention cases only. The preoperative survey will be administered after viewing the model but before the operation, and the postoperative survey will be administered after the operation. These surveys are described in Appendix B

VI. RESEARCHERS AND RESPONSIBILITIES

a. Principal Investigator (PI): One PI from each participating Site.
  - i. Overall Site point person for study.
  - ii. Coordinates support for study with other Site participants, including Research Coordinator and surgeon users.
  - iii. Primary interface with the Site's IRB.

b. **Research Coordinator ("RC").** One RC from each participating site. A researcher, fellow, resident or student identified and recruited by the PI. Key activities:
  - i. Identify upcoming cases to be included in the study and assign cases to control group or intervention group using block randomization.
  - ii. Identify, collect and manage:
    1. The MRIs from which the models will be created
    2. Data regarding Control Cases ("***Control Case Data***") and intervention cases ("***Intervention Case Data***")
  - iii. Deidentify each source MRI using deidentifying software (or ensures that Radiology deidentifies the image prior to delivery to the RC); deliver the deidentified image to Ceevra along with a unique identifier.
  - iv. Coordinate with surgeon users to ensure that both the model and the underlying MRI are reviewed prior to intervention cases.
  - v. Assist with administration of the surgeon surveys

c. **Surgeon Users.** Estimated between one and eight surgeon users (inclusive of the PI) from each site. Key activities:
    i. Oversee receipt of informed consents from their patients participating in the study.
    ii. Provide the mobile phone used for viewing the models
    iii. Before each intervention case, review the source MRI and then review the associated model for purposes of case planning.
    iv. Participate in surgeon surveys.

VII. MODEL CREATION KEY PERSONNEL

a. **Russ Yoshinaka, CEO**. Overall Ceevra point person for study.
b. **Ken Koster, CTO.**
    i. Oversees the creation of the models from the MRIs.
    ii. Oversees Ceevra software application and infrastructure.
    iii. Primary point of contact for user technical support issues.

VIII. DATA COLLECTION AND SECURITY PLAN

a. **Protocol for Case Data.** Case data will be extracted from the site's EHR system, and the surveys. It will be tracked via two files:
    i. The key file will include patient information (listed below) and a unique identifier. This file will be password protected, maintained solely at the site, and accessible by site researchers only.
        1. Medical Record Number (MRN)
        2. Surgeon Number
        3. MRI ID #
        4. Operation Date
        5. Unique Identifier
    ii. The data file will exist either as a data repository (Open Clinica) or as a on-site file, per site preference, and will include the unique identifier and outcomes data, but no PHI. This file will be password protected.
b. **Protocol for Model Creation and Data Security**
    i. PI/RC identifies upcoming intervention cases for which models are to be created.
    ii. PI/RC obtains source MRI, deidentifies the same using deidentifying software (or ensures that radiology deidentifies the image prior to delivery to the PI/RC), and delivers it to Ceevra along with a unique identifier.
    iii. Ceevra creates model from the deidentified MRI. Once completed:
        1. Delivers the model back to PI/RC, along with the unique identifier.
        2. Notifies applicable surgeon user (with copy to PI and RC) that model is available for viewing through the mobile app.
    iv. Prior to the operation, PI/RC contacts applicable surgeon user to ensure viewing of model along with source MRI.
    v. The use of the model will be an additional step undertaken by the surgeons in preparation for surgery and during the operation itself and will not impact the surgeons' standard review of subject images.

IX. TRAINING, MEASURING & COMMUNICATING

a. Training with surgeon users on accessing/using mobile app and models. Each surgeon will be trained either in person or via video call. During the training, the

surgeon will be trained how to access the models from their mobile phones and how utilize several viewing features such as showing/hiding anatomical parts; zooming in/out on the model; and viewing the on the surgical robot.

b. Kickoff Meeting (in-person meeting at medical center):
   i. Introduce participants
   ii. Outline study processes, timeline and objectives
c. Monthly virtual meetings
   i. Report study results to date
   ii. Gather feedback – both on specific items and general discussion
d. Surgeon Surveys
e. Study Conclusion Meeting (in-person): Final study results

Appendix A

Study Covariates

1. Patient parameters
    a. Age
    b. Race
    c. Surgeon ID and tier
    d. MRI Fusion Biopsy used
    e. Preop and postop Gleason score
    f. Preop PSA
    g. Preop SHIM score
    h. Preop use of erectile aid
    i. Prostate volume
    j. Number of masses
    k. Mass size
    l. Extracapsular extension
    m. Tumor location
    n. Tumor orientation
    o. PIRADS score
    p. Perineural invasion
    q. SV invasion
    r. Risk category
    s. Clinical stage
    t. Oncologic stage
    u. Number of positive biopsy cores
    v. Use of erectile aids (pre and postoperatively)
2. Surgical technique
    a. Node dissection template, if performed
    b. Assistant port used
    c. SV dissection approach
3. Operative Parameters
    a. Total operative time
    b. Blood Loss (cc)
    c. Conversion to open (y/n)
    d. Operative complication (y/n)
    a. Hospital stay (days)
    b. Mortality (y/n)
    c. Readmission (y/n)

Appendix B
Surgeon Pre- and Postoperative Survey

Preoperative Survey (after viewing MRI but before viewing model)

1) Do you plan to perform nerve sparing (none, right, left, bilateral)
2) Do you plan to perform bladder neck preservation (yes, no)

Preoperative Survey (after viewing MRI and model)

1) Do you plan to perform nerve sparing (none, right, left, bilateral)
2) Do you plan to perform bladder neck preservation (yes, no)

Postoperative Survey

1) How would you rate your ability to see and define the area of cancer in the MRI (Likert)
2) How would you rate your ability to see and understand the relationship between the mass and the adjacent structures in the MRI (Likert)
3) How would you rate your ability to see and define the area of cancer in the model (Likert)
4) How would you rate your ability to see and understand the relationship between the mass and the adjacent structures in the model (Likert)
5) Were there any anatomical structures that you were able to see (or see better) in the model as opposed to the MRI (yes, no)
6) If answer to the question above is yes, please explain (free text)
7) Were there any structures or details that you could see in the source MRI that were either missing from, or appeared to be inaccurate in the model (yes, no)
8) If answer to the question above is yes, please explain (free text)
9) Did you modify your preoperative plan in any way as a result of what you saw in the model (yes, no)
10) If answer to the question above is yes, please describe how (free text)
11) Did you perform nerve sparing (yes, no)
12) How much did the model aid you in performing nerve sparing, if performed (Likert)
13) Did you perform bladder neck preservation (yes, no)
14) How much did the model aid you in performing nerve sparing, if performed (Likert)
