# Supplementary material for: Trifecta Outcomes After Use of 3-Dimensional Digital Models for Planning of Robotic Prostatectomy: A Secondary Analysis of a Randomized Clinical Trial
Source: JAMA Netw Open. 2024 Sep 16;7(9):e2434143. doi: 10.1001/jamanetworkopen.2024.34143 (PMC11406400; doi:10.1001/jamanetworkopen.2024.34143)
Supplement: Supplement 2. — eFigure. Positive Margin Rate Between Study Group by Grade Group and Pathological Stage [file jamanetwopen-e2434143-s002.pdf]

Supplementary Online Content

Shirk JD, Reiter R, Wallen EM, et al. Trifecta outcomes after use of 3-dimensional digital models for planning of robotic prostatectomy: a secondary analysis of a randomized clinical trial. *JAMA Netw Open*. 2024;7(9):e2434143. doi:10.1001/jamanetworkopen.2024.34143

eFigure. Positive Margin Rate Between Study Group by Grade Group and Pathological Stage

This supplementary material has been provided by the authors to give readers additional information about their work.

© 2024 Shirk JD et al. *JAMA Network Open.*

eFigure. Positive Margin Rate Between Study Group by Grade Group and Pathological Stage

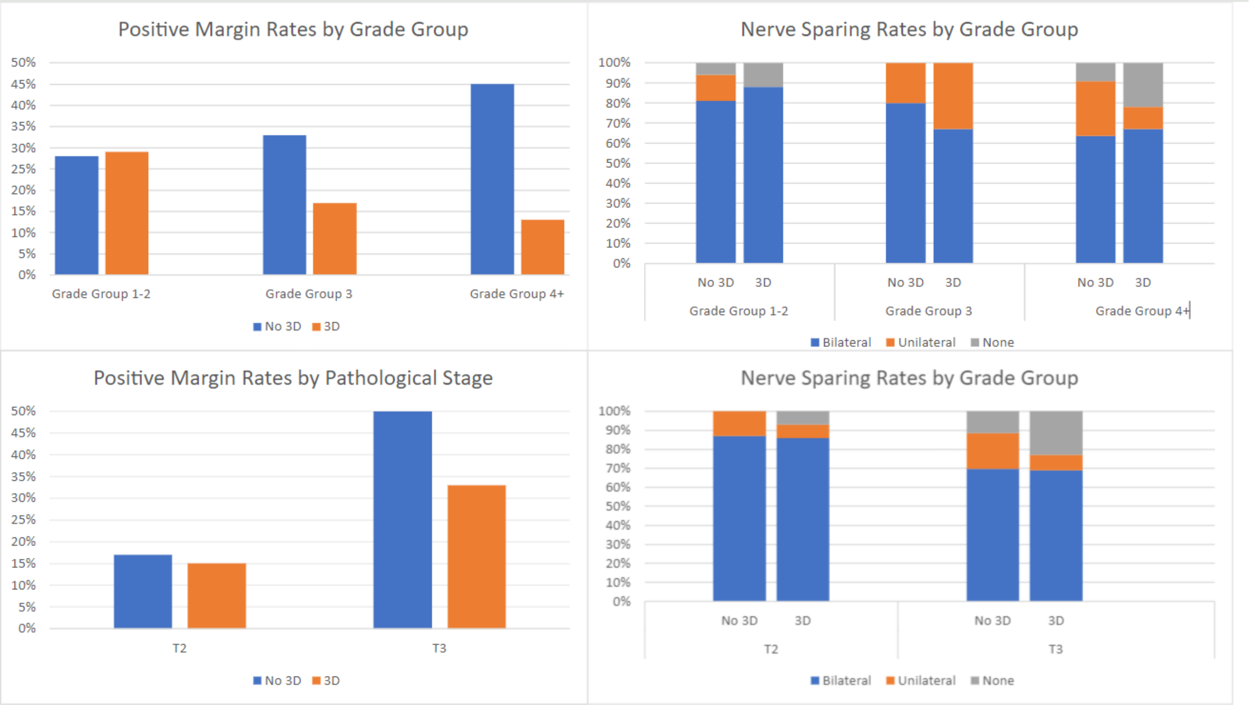

© 2024 Shirk JD et al. *JAMA Network Open.*
