# Supplementary material for: Trifecta Outcomes After Use of 3-Dimensional Digital Models for Planning of Robotic Prostatectomy: A Secondary Analysis of a Randomized Clinical Trial
Source: JAMA Netw Open. 2024 Sep 16;7(9):e2434143. doi: 10.1001/jamanetworkopen.2024.34143 (PMC11406400; doi:10.1001/jamanetworkopen.2024.34143)
Supplement: Supplement 3. — Data Sharing Statement [file jamanetwopen-e2434143-s003.pdf]

Data Sharing Statement

Shirk. Trifecta Outcomes After Use of 3-Dimensional Digital Models for Planning of Robotic Prostatectomy. *JAMA Netw Open*. Published September 19, 2024.
doi:10.1001/jamanetworkopen.2024.34143

Data
Data available: No

Additional Information
Explanation for why data not available: Per individual site agreements, data not available for sharing
